# A divergent strain of melon chlorotic spot virus isolated from black medic (*Medicago lupulina*) in Austria

**DOI:** 10.1186/s12985-019-1195-8

**Published:** 2019-07-05

**Authors:** Yahya Z. A. Gaafar, Katja R. Richert-Pöggeler, Angelika Sieg-Müller, Petra Lüddecke, Kerstin Herz, Jonas Hartrick, Yvonne Seide, Heinrich-Josef Vetten, Heiko Ziebell

**Affiliations:** 1 Julius Kühn Institute, Institute for Epidemiology and Pathogen Diagnostics, Messeweg 11-12, 38104 Braunschweig, Germany; 2 Im Spargelfeld 1, 38162 Cremlingen, Germany

**Keywords:** High throughput sequencing, Melon chlorotic spot virus, Segmented virus, *Medicago sativa*, *Pisum sativum*, *Vicia faba*

## Abstract

A tenuivirus, referred to here as JKI 29327, was isolated from a black medic (*Medicago lupulina*) plant collected in Austria. The virus was mechanically transmitted to *Nicotiana benthamiana*, *M. lupulina*, *M. sativa*, *Pisum sativum* and *Vicia faba*. The complete genome was determined by high throughput sequencing. The genome of JKI 29327 consists of eight RNA segments closely related to those of melon chlorotic spot virus (MeCSV) isolate E11–018 from France. Since segments RNA 7 and 8 of JKI 29327 are shorter, its genome is slightly smaller (by 247 nts) than that of E11–018. Pairwise comparisons between the predicted virus proteins of JKI 29327 and their homologues in E11–018 showed aa identities ranging from 80.6 to 97.2%. Plants infected with E11–081 gave intermediate DAS-ELISA reactions with polyclonal antibodies to JKI 29327. Since JKI 29327 and E11–018 appear to be closely related both serologically and genetically, we propose to regard JKI 29327 as the black medic strain of MeCSV. To our knowledge, JKI 29327 represents the second tenuivirus identified from a dicotyledonous plant. Serological and molecular diagnostic methods were developed for future detection.

## Main text

Members of the genus *Tenuivirus*, family *Phenuiviridae*, are plant viruses that possess non-enveloped filamentous particles and a genome consisting of four to eight single-stranded RNA segments with negative or ambisense polarity. The thin filamentous particles consist of ribonucleoprotein (RNP) complexes, measuring 3–10 nm in diameter and with lengths proportional to the sizes of the RNAs they contain. Based on the RNA sizes, the particles may appear as small, large or even branched circles [1, 2]. Tenuivirus RNAs are neither capped at their 5′ end nor polyadenylated at the 3′ end. The nucleotide sequences of the 5′ and 3′ ends of each segment are complementary [1]. Tenuiviruses are known to be transmitted by planthoppers or by mechanical means albeit with difficulty [1]. According to the International Committee on Taxonomy of Viruses (ICTV), seven virus species are currently assigned to the genus *Tenuivirus*: *Echinochloa hoja blanca virus* (EHBV), *Iranian wheat stripe virus* (IWSV), *Maize stripe virus* (MSpV), *Rice grassy stunt virus* (RGSV), *Rice hoja blanca virus* (RHBV), *Rice stripe virus* (RSV) and *Urochloa hoja blanca virus* (UHBV). In addition, three more species have been proposed and are pending recognition by ICTV: melon chlorotic spot virus (MeCSV), Ramu stunt virus (RmSV) and wheat yellow head virus (WYHV) [2–4]. The natural host range of tenuiviruses is typically restricted to monocots of the *Poaceae* family causing yield losses in important food crops such as rice (*Oryza sativa* L.) and maize (*Zea mays* L.) [5]. The recent identification of MeCSV from melon (*Cucumis melo*) in France represents the first report of a tenuivirus naturally infecting a dicotyledonous plant [2].

**Fig. 1:**
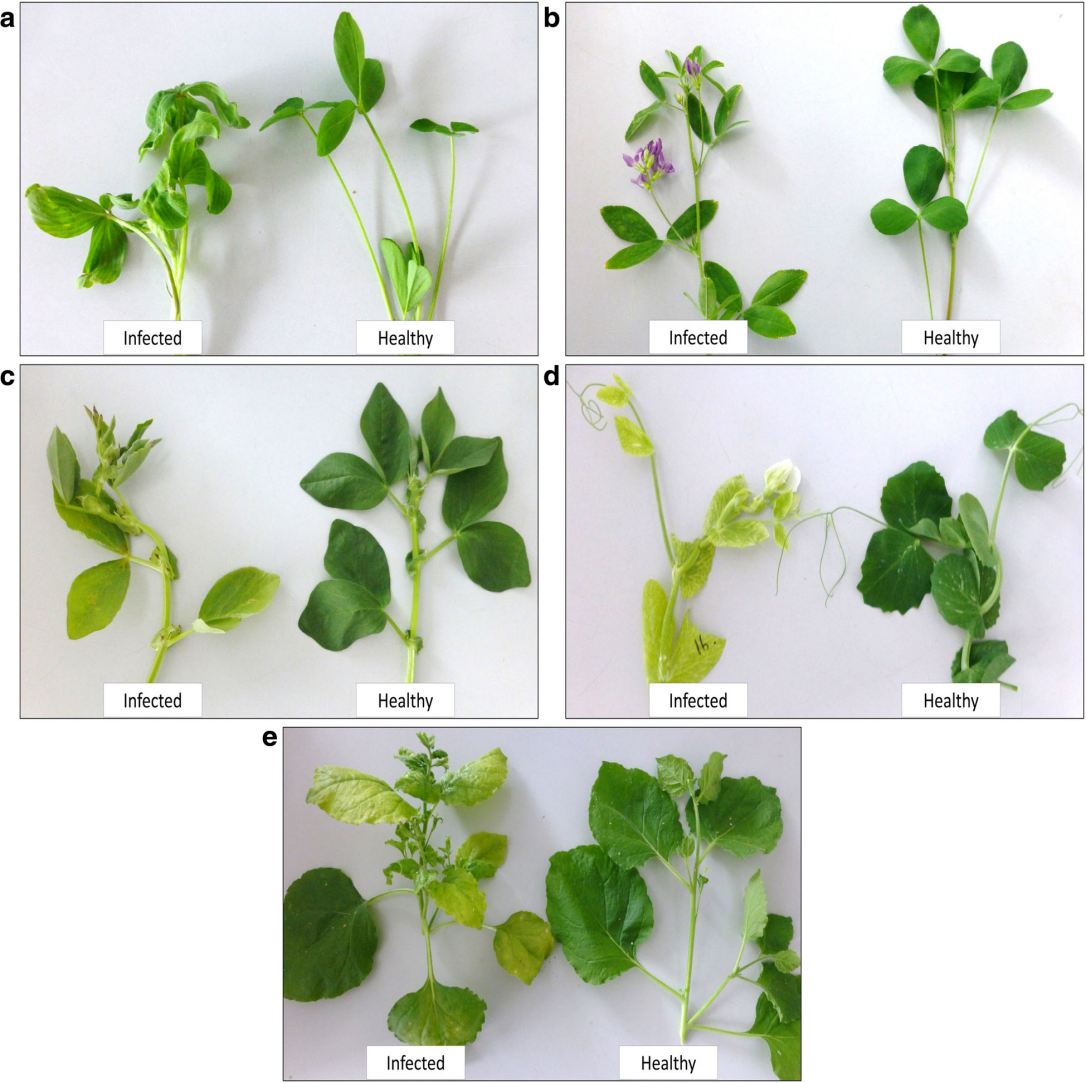
Plants infected with the black medic tenuivirus (JKI 29327): (**a**) *Medicago lupulina*, (**b**) *M. sativa*, (**c**) *Vicia faba*, (**d**) *Pisum sativum* and (**e**) *Nicotiana benthamiana*

In 2011, a black medic (*Medicago lupulina* L.) plant showing virus-like symptoms was collected in Stadl-Paura, Austria, but the symptoms were not recorded at the time. The sample was sent to Julius Kuehn Institute for analysis. Electron microscopy revealed the presence of RNP that appeared to resemble disassembled rhabdovirus particles [6, 7]. However, polyclonal antibodies JKI-1607 raised against alfalfa-associated nucleorhabdovirus (AaNV) [7] failed to react with this virus in DAS-ELISA. The virus was transmitted mechanically as described in [7] to *Nicotiana benthamiana*, *M. lupulina*, *M. sativa*, *Pisum sativum* and *Vicia faba*, and was referred to as JKI 29327. Three weeks post inoculation, *N. benthamiana* plants showed systemic mottling, slight vein clearing and top leaf curling, whilst *M. lupulina* and *M. sativa* plants showed systemic vein clearing. *P. sativum* plants showed systemic vein clearing and severe yellowing and *Vicia faba* showed systemic mottling, yellowing and leaf rolling (Fig. 1). The virus particles of JKI 29327 were partially purified from infected *N. benthamiana* and used for antiserum production as described before [7]. The antibodies (JKI-1608) were used for DAS-ELISA analysis of sap-inoculated plants and confirmed infection of symptomatic plants.

**Fig. 2:**
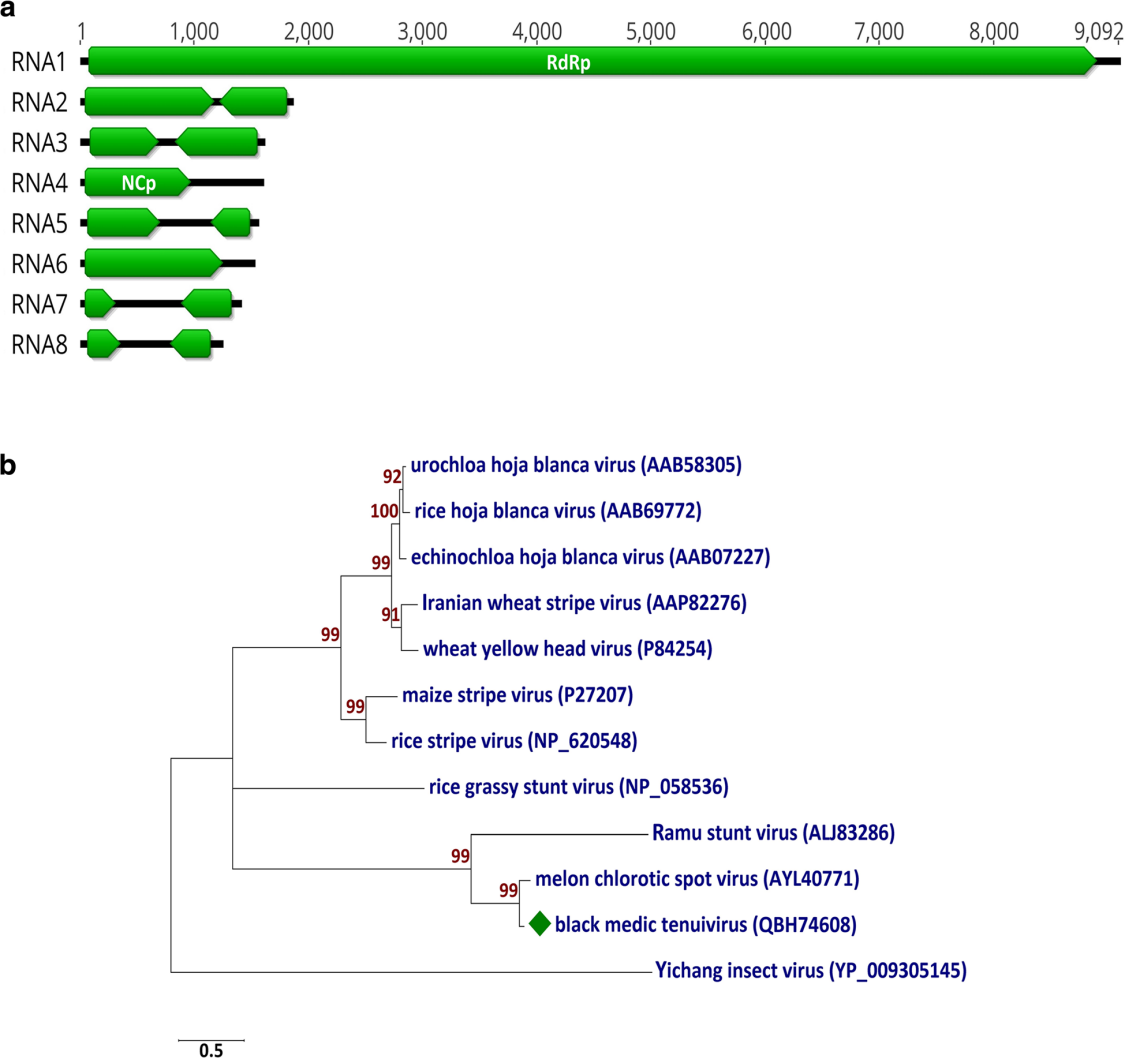
(**a**) Graphical representation of the genome of the black medic tenuivirus isolate JKI 29327. (**b**) Maximum-likelihood (ML) phylogenetic tree (using Jones-Taylor-Thornton (JTT) model) based on the amino acid sequence alignments of the nucleocapsid proteins (NCp) of JKI 29327 and members of the *Tenuivirus* genus. The GenBank accession nos. are in brackets. Yichang insect virus (genus *Goukovirus*) was used as an outgroup sequence. Numbers on branches indicate the bootstrap percentages (1000 replicates, only values ≥50% are shown) and the scale bar represents a genetic distance of 0.5

For determination of the complete genome sequence of JKI 29327, total RNA was extracted from infected *N. benthamiana* using innuPREP RNA Mini Kit (Analytik Jena AG) followed by ribosomal RNA depletion using the RiboMinus Plant kit (Invitrogen). The ribo-depleted RNA was used for high throughput sequencing (HTS) on a MiSeq (v3) platform (2 × 301) as described before [7]. A total of 2,056,956 reads were obtained. The raw reads were quality trimmed and size filtered using Geneious Prime (v. 2019.0.3) (Biomatters Limited). The reads were then de novo assembled using Geneious assembler. A total of 53,651 contigs were generated and used for Blastn and Blastx search using virus/viroid databases on NCBI. Fifty-eight contigs shared nucleotide (nt) sequence identities (from 73.5 to 90.6%) and amino acid (aa) sequence identities from 63.8 to 97.2% to MeCSV. No other virus sequences were detected. The reference sequences of MeCSV (NC_040448 to NC_040455) were used to map the black medic tenuivirus sequences. The complete genome sequence of JKI 29327 (containing eight segments (Fig. 2a)) was assembled (19,805 nt; accession nos. MK450511 to MK450518) but segment RNA7 and RNA8 were 94 nt and 177 nt shorter than the genome of the isolate E11–018 of MeCSV. Analysis of each segment showed the presence of conserved nt sequences which can also be observed in other tenuiviruses (ACA CAA AGU C at the 5′ end with its complementary sequence UGU GUU UCA G at the 3′ end). Eight primers pairs were designed using Primer 3 (2.3.7) tool in Geneious (Table 1) to confirm the physical presence of all eight viral segments using RT-PCR (OneTaq One-Step RT-PCR Kit; NEB) [8] on fresh RNA extracts from *N. benthamiana*. The amplicons were gel-purified using Zymoclean Gel DNA Recovery Kit (Zymo Research) and Sanger sequenced; sequence analyses of these amplicons showed that they were 100% identical to the corresponding segment sequences obtained by the HTS analysis and thus confirmed the presence of each individual viral segment.

**Table 1:** The genome characteristics of melon chlorotic spot virus (MeCSV) isolate (JKI 29327) from Austria; nt and aa sequence identities of the 8 RNA segments compared to the respective homologous regions in the genome of MeCSV isolate E11–018 from France and the list of primers used for segment identification

Genome segments					Intergenic region (IR)		Predicted proteins				
Type	Length (nt)	Percent nt identity to MeCSV E11–018	PCR primers used for JKI 29327		Length (nt)	Percent nt identity to MeCSV E11–018	ORF	Putative functions	Length (aa)	Size (kDa)	Percent aa identity to MeCSV E11–018
			Name	Sequence							
RNA1	9092	82.4	HZ-603	5′ ACA GAA GTG GAA TGG GCT GG 3’	NA	NA	ORF1	RNA-dependent RNA polymerase	2940	340	92.1
			HZ-604	5′ GCA ACA CCC TCA TCA CTC CA 3’							
RNA2	1847	84.6	HZ-605	5′ AGC TCA GTA ACC GGA ACT GC 3’	50	100	ORF2a	no match	373	43.6	87.7
			HZ-606	5′ CGC AAT AGC AGG GTC CAG AT 3’			ORF2b	no match	196	43.7	87.3
RNA3	1598	85.8	HZ-607	5′ TGG TGC CAG AAG GAA AGG AC 3’	157	72.6	ORF3a	no match	195	23.2	93.3
			HZ-608	5′ GGC AAT GCC TCA CAA TCG TC 3’			ORF3b	no match	235	27.6	89.8
RNA4	1591	78.3	HZ-609	5′ AAG TAA GGG CAG GCT GAA CC 3’	NA	NA	ORF4	nucleocapsid protein	305	33.6	88.2
			HZ-610	5′ AGG CTT TCT GCT AAG TGG GC 3’							
RNA5	1547	81.7	HZ-611	5′ GAA CTG TAC CGC TGA TGG GT 3’	446	68.3	ORF5a	no match	210	23.2	93.3
			HZ-612	5′ CTT TGG TCT GGA GCT GTG CT 3’			ORF5b	no match	114	13	95.6
RNA6	1509	84.7	HZ-613	5′ CGC ATC CTG AAT CCC ATC TCT 3’	NA	NA	ORF6	no match	400	45.5	91.2
			HZ-614	5′ GCT GGC ATC ACT AGA CGG AT 3’							
RNA7	1392	73.2	HZ-615	5′ ATC AGG TGT TAG CTG GCC AC 3’	586	55.6	ORF7a	no match	84	9.8	90.5
			HZ-616	5′ TAA CCA CCT TCC CTG CTG TG 3’			ORF7b	no match	143	15.9	80.6
RNA8	1229	68.7	HZ-617	5′ ACC CTA AGT GGA TCC GAG GT 3’	446	58.3	ORF8a	no match	93	10.9	94.8
			HZ-618	5′ AGT TCC AAG TTG CCC TGC TT 3’			ORF8b	no match	115	12.9	97.2

To predict the open reading frames’ (ORF) functions, the translation of each ORF was used to search for conserved domains on NCBI’s conserved domain database (CDD v 3.16) [9]. Only two ORFs matched with entries in the database, i.e., RNA1-ORF1 with *Bunyavirus* RNA-dependent RNA polymerase (accession no.: cl20265) and RNA4-ORF1 with *Tenuivirus*/*Phlebovirus* nucleocapsid protein (accession no.: cl05345) (Table 1). Pairwise alignments for the different regions of each segment of JKI 29327 were performed with their homologous sequences of MeCSV using CLUSTALW (Table 1) [10]. The genome components of JKI 29327 shared nt identities ranging from 68.7 to 85.8% with those of the MeCSV isolate E11–0188 (Table 1). The proteins potentially encoded by JKI 29327 and E11–018 shared aa sequence identities ranging from 80.6 to 97.2% (Table 1). A maximum-likelihood (ML) phylogenetic tree was generated using MEGA7 (7.0.26) (Jones-Taylor-Thornton (JTT) model) [11] for comparing the aa sequence of nucleocapsid proteins of JKI 29327 and other tenuiviruses. This showed a tight clustering of JKI 29327 with the MeCSV nucleoprotein (Fig. 2b). Additionally, RNA segments 7 and 8 respectively have shorter intergenic regions (IR) (586 and 446 nt) compared with those (680 and 623 nt) on the homologous RNAs of E11–018. The nt sequence identities between these IR regions of RNA7 and RNA8 are 55.6 and 58.3%, respectively. The results indicate that JKI 29327 is closely related to but distinct from the MeCSV isolate E11–018.

**Fig. 3:**
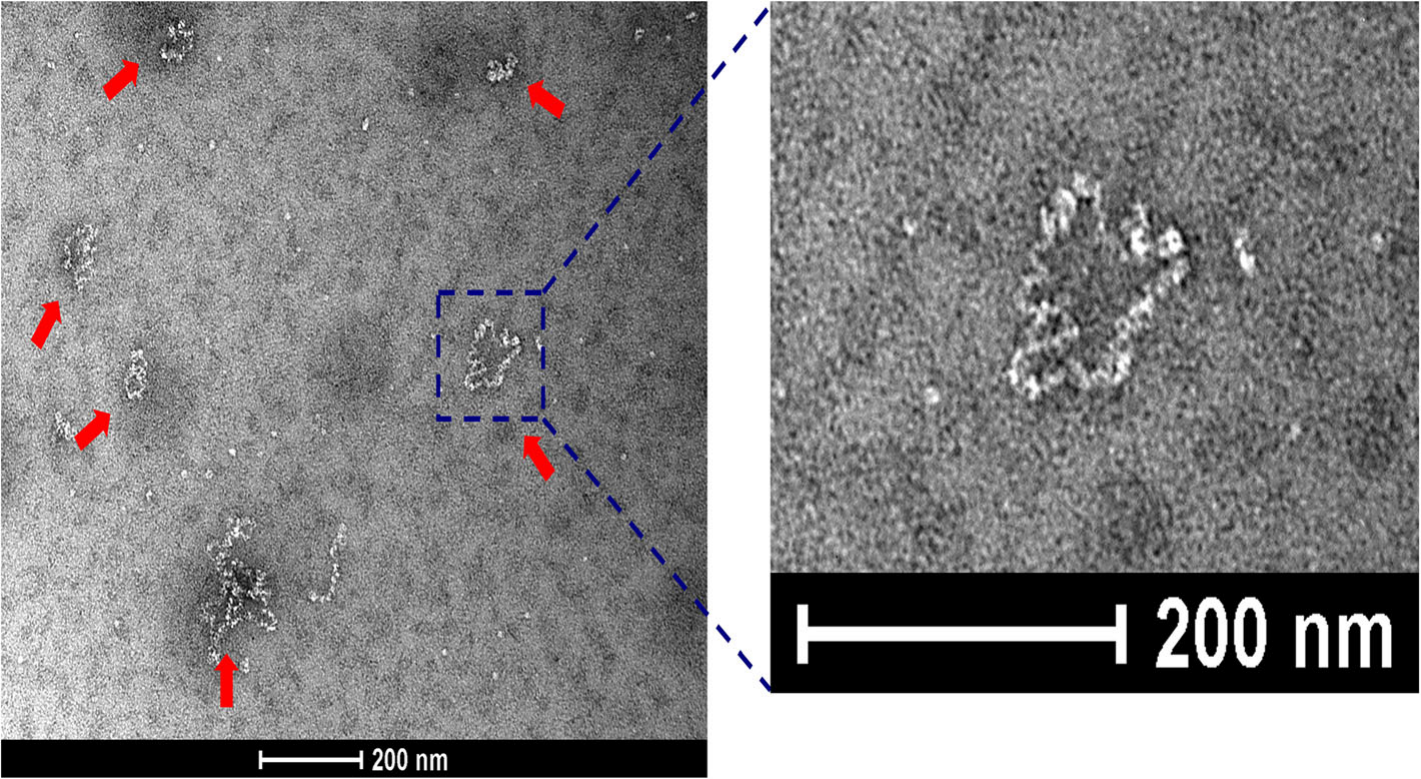
Electron micrograph of a purified ribonucleoprotein preparation showing tenuivirus-like circular filamentous ribonucleoproteins (RNP) of different sizes (red arrows)

For additional confirmation, purified RNP preparations of JKI 29327 were again examined by electron microscopy and shown to contain tenuivirus-like circular filamentous particles representing the individual genome segments (Fig. 3).

To assess the serological relationship between JKI 29327 and E11–018, *N. benthamiana* and *Physalis floridana* leaves infected with the MeCSV isolate E11–018 (kindly provided by Dr. C. Desbiez) were tested in DAS-ELISA using the JKI 1608 antibodies to JKI 29327. The latter gave strong (A_405 nm_ values: > 2.0) reactions with JKI 29327 (in four different plant spp.) and intermediate reactions (A_405 nm_: 1.0 to 2.0) with E11–018, indicating that the serological relationship between these two isolates is close. Additionally, JKI 29327 was mechanically inoculated to melon cv. Védrantais (kindly provided by Dr. C. Desbiez). The plants showed chlorotic spots only on inoculated leaves and tested positive in DAS-ELISA with the JKI 1608 antibodies. Whilst JKI 29327 could be detected in inoculated leaves, no systemic infection was observed (data not shown).

The species demarcation criteria of ICTV for the genus *Tenuivirus* suggest that a new species should be considered when (i) the aa sequence identities between any corresponding gene products is below 85%; (ii) the nt sequence identities between corresponding IRs is below 60%; (iii) there are different sizes and/or numbers of genomic components; (iv) there are differences in host range; (v) the vectors are different [1]. For certain tenuiviruses, it has been difficult to decide whether they belong to the same or different species because all the five criteria are not always met [1]. For example, RHBV, EHBV and UHBV have different vectors, different hosts, different sizes and numbers of RNA segments and the nt sequence identity of their IR is < 60%. Yet, the four protein homologs on their RNA3 and RNA4 are 90% identical in aa sequences.

The black medic tenuivirus isolate JKI 29327 fulfils three out of these five criteria. Firstly, its ORF2 of RNA7 shares 80.6% aa identity with its homologue in the E11–081 genome. Secondly, the IRs of both RNA7 and RNA8 share < 60% nt identities with those of E11–081. Thirdly, the overall genome size of JKI 29327 is 247 nt shorter than that of E11–081. Based on these three criteria, the black medic virus should be considered a new species. However, although the host range was not studied in full detail, both JKI 29327 and E11–081 infected members of the *Fabaceae*, the *Cucurbitaceae* and the *Solanaceae* families under experimental conditions. Moreover, these two isolates appear to be serologically closely related when tested with the JKI 1608 antibodies. Small differences in size, particularly in the intergenic regions, are common and can be observed between isolates of RSV [12, 13]. Also, segment RNA 7 of MeCSV E11–018 was shown to present size heterogeneity due to indels in the intergenic region [2]. Furthermore, only one protein out of 13 was below the 85% identity threshold. Therefore, we propose that the black medic isolate from Austria is a strain of MeCSV and is referred to accordingly as black medic strain of MeCSV. Further studies are required to identify possible natural hosts and insects that may act as vectors of both JKI 29327 and E11–081. Moreover, there is a need to compare the experimental and natural host ranges of the two MeCSV strains. The antiserum obtained in this study will help to monitor prevalence and geographic distribution of MeCSV as well as its agronomic impact on crop plants (e.g., melons, legumes). Furthermore, it is important to study the function of the virus proteins that have been predicted in silico.

## Data Availability

Raw sequence data are available in the Sequence Read Archive (SRA) under BioSample accession number SAMN11974730, BioProject accession number PRJNA524397​.

## Abbreviations

aa: amino acid
DAS-ELISA: double antibody sandwich enzyme-linked immunosorbent assay
EHBV: echinochloa hoja blanca virus
EM: electron microscopy
HTS: high throughput sequencing
ICTV: International Committee on Taxonomy of Viruses
IR: intergenic regions
JKI: Julius Kuehn Institute
MeCSV: melon chlorotic spot virus
ML: maximum-likelihood
MSpV: maize stripe virus
NCp: nucleocapsid protein
nt: nucleotide
RdRp: RNA-dependent RNA polymerase
RHBV: rice hoja blanca virus
RmSV: Ramu stunt virus
RNP: ribonucleoprotein
RSV: rice stripe virus
RT-PCR: reverse transcription polymerase chain reaction
UHBV: urochloa hoja blanca virus
WYHV: wheat yellow head virus
